# Enhancement of postharvest longan fruit quality through chitosan (CTS)-induced modulation of energy and proline metabolism

**DOI:** 10.1038/s41598-025-18761-w

**Published:** 2025-09-29

**Authors:** Xinmin Lv, Yulian Li, Jiaying Chen, Shilian Huang, Junbin Wei, Shiya Xu, Dongliang Guo, Yan Zhou

**Affiliations:** 1 Institute of Fruit Tree Research, Guangdong Academy of Agricultural Sciences, Guangzhou, 510640 China; 2 Key Laboratory of South Subtropical Fruit Biology and Genetic Resource Utilization , Ministry of Agriculture and Rural Affairs, Guangzhou, China 510640; 3 Guangdong Provincial Key Laboratory of Science and Technology Research on Fruit Tree , Guangzhou, China 510640; 4 Life Science and Technology School , Lingnan Normal University , Zhanjiang, China 524048; 5 GuangDong Technology Innovation Center of Tropical Characteristic Plant Resource Development, Lingnan Normal University, Zhanjiang, 524048 China; 6 Zhanjiang Key Laboratory of Tropical Characteristic Plant Technology Development , Lingnan Normal University , Zhanjiang, China 524048

**Keywords:** Preservative, *Storage stability*, *Metabolic regulation*, *Antioxidant*, Physiology, Plant sciences

## Abstract

Chitosan (CTS), a biologically safe preservative, is crucial for post-harvest treatment of fruit and vegetables, yet its regulatory mechanisms remain poorly understood. Our research on various CTS concentrations in longan fruit post-harvest showed that 1.5% CTS curtailed Weight loss and malondialdehyde content while enhancing soluble solid content, carotenoids, ascorbic acid, total phenols, and flavonoids. Transcript-level analysis revealed that 1.5% CTS maintains longan fruit quality by modulating energy and amino acid metabolism pathways. Further evaluations demonstrated increased energy charge, adenosine triphosphate (ATP), and adenosine diphosphate (ADP) levels, alongside elevated activities of H^+^-ATPase, Ca^2+^-ATPase, cytochrome C oxidase, and succinate dehydrogenase in CTS-treated fruit. CTS also upregulated genes like *H*^*+*^*-ATPase1*, *SDH1*, *SDH6*, *CCO5b-2*, and *CCO6b-2*, boosted proline content, and enhanced activities of proline-metabolizing enzymes Δ1-pyrroline-5-carboxylic acid synthase (P5CS) and ornithine-ծ-aminotransferase (OAT), while reducing pyruvate dehydrogenase (PDH) activity. These results suggest that CTS effectively modulates gene expression and enzymatic functions related to energy and proline metabolism, enhancing the preservation of longan fruit freshness during storage.

**Supplementary Information:**

The online version contains supplementary material available at 10.1038/s41598-025-18761-w.

## Introduction

Longan, a significant fruit crop, is widely cultivated in Southern China^1^. Notably rich in carbohydrates, proteins, vitamins, and trace minerals, longan fruit embodies essential nutrients^2^. Its ripening period aligns with summer’s peak temperatures, posing considerable challenges to post-harvest preservation^2^. At ambient conditions, longan fruit exhibit a brief shelf Life of merely 2~ 3 days, prone to swift spoilage characterized by pericarp browning, pulp liquefaction, and an enhanced susceptibility to pathogens, thereby severely affecting their transportability and marketability^3^. However, extensive research indicates that employing antioxidants^4^, organic acids^5^, adenosine triphosphate (ATP)^3^, thermal treatments^6^, and edible coatings^1,7^ can markedly improve postharvest durability, thus preserving fruit quality effectively. However, the application of these exogenous substances often encounters various drawbacks in production settings, highlighting the necessity for exploring safe, efficient, environmentally friendly, and convenient preservatives or preservation methods for post-harvest fruit treatment.

Chitosan (CTS), a high-molecular-weight cationic polysaccharide, is widely recognized for its biosafety and efficacy as a preservative in the post-harvest treatment of fruit and vegetables. Its use as a coating minimizes water loss and adjusts O_2_ and CO_2_ permeability, thereby improving storage quality and reducing disease incidence^7–9^. CTS also boosts the production of lignin, phenolics, and antioxidative agents in plants, enhancing their defense against pathogens and further ensuring superior preservation^10,11^. This effectiveness is evidenced in the enhanced storage quality of blueberries, strawberries, and pitaya, marked by increased levels of total phenolics, ascorbic acid, glutathione, and flavonoids^12,13^. Despite the widespread use of CTS as a preservative in the preservation of fruit and vegetables, research into the regulatory mechanisms through which it preserves these foods remains limited.

In post-harvest fruit and vegetables, quality maintenance is significantly influenced by the combined effects of energy and proline metabolism, which are central to the regulation of cellular physiological activities^9,14,15^. During the energy metabolism phase, ATP hydrolases decompose ATP into adenosine diphosphate (ADP), inorganic phosphate (Pi), and energy, utilizing the high-energy phosphoanhydride bond in a process that efficiently manages energy storage and release^14,16^. This includes key enzymes such as H^+^-ATPase, Ca^2+^-ATPase, and Mg^2+^-ATPase. The energy status within cells, quantified as the EC based on the levels of ATP, ADP, and AMP, is crucial for assessing the health of post-harvest fruit. Higher enzymatic activity, indicative of robust energy metabolism, leads to increased ATP and EC levels, enhancing cellular stress resistance and reducing the risk of membrane structure disruption^9,17^. Conversely, an energy imbalance can trigger faster quality decline, accelerating senescence and increasing the likelihood of fruit spoilage^18,19^. Thus, managing ATP levels and EC is vital for supporting the metabolic and cellular processes essential for extending the shelf life of post-harvest fruit^3,20^.

Proline, a critical osmoregulatory compound, is essential in the adaptive responses of fruit and vegetables to various environmental stresses, including low-temperature storage conditions. Accumulation of proline during post-harvest storage plays a pivotal role in reducing chilling injury and enhancing cold tolerance, as demonstrated in a range of produce^21^. A key to this process is maintaining high-energy cellular states, which promotes proline production, thereby preserving post-harvest quality and prolonging shelf life. For instance, eggplants exposed to low temperatures not only show increased chilling damage but also a corresponding rise in proline levels, indicating a stress response^22^. Similarly, treatments with methyl jasmonate and γ-aminobutyric acid enhance the activity of enzymes involved in proline synthesis—namely, ornithine-ծ-aminotransferase (OAT), Δ1-pyrroline-5-carboxylic acid synthase (P5CS), and pyruvate dehydrogenase (PDH)—in peach and loquat fruit, facilitating proline accumulation and improving cold tolerance^23^. This effect is also observed in Hami melons treated with oxalic acid under cold storage, where proline content increases with prolonged low-temperature exposure^24^. Similar observations in the proline dynamics have been reported in studies on cucumbers^25^, bananas^26^, and peaches^27^, underscoring the compound’s universal role in stress mitigation.

The majority of previous research on CTS as a preservative for fruit and vegetables has centered on its effects against post-harvest pathogens, largely overlooking its potential impacts on these crucial metabolic pathways. However, while numerous studies have highlighted the efficacy of CTS in preserving fruit and vegetables, the underlying mechanisms of its preservative effects are still largely unexplored. To address this gap, our study investigates the role of CTS in enhancing the post-harvest quality of longan fruit. Through a combination of transcriptomic and biochemical analyses, we aim to uncover how CTS influences energy and proline metabolism pathways, with the goal of extending shelf life and reducing decay. Our research seeks to establish a theoretical basis and reference for the development of preservation technologies that target these metabolic processes. Numerous studies have highlighted the critical role of maintaining a balance between energy and proline metabolism for preserving the quality of post-harvest fruit.

## Materials and methods

### Materials and treatment

Mature ‘Chuliang’ longan fruit (*Dimocarpus longan* L.) were harvested from a commercial farm in Haikou, Hainan Province, China. A total of 600 fruit were carefully sorted and thoroughly examined for quality, ensuring the absence of flaws and maintaining consistency. The longans were washed with a 0.05% sodium hypochlorite solution and allowed to air dry naturally. They were then subjected to five different treatments: (1) Control: distilled water; (2) 0.5% CTS; (3) 1.0% CTS; (4) 1.5% CTS; and (5) 2.0% CTS, with each treatment consisting of 120 fruit samples. All treatment solutions included Tween-80 at a concentration of 1:1,000 (v/v). The longan fruit were soaked in the treatment solutions for 5 min and then air-dried. Subsequently, they were stored at 25 ± 1 °C with a relative humidity of 80–85%. Longan fruit flesh tissues were collected on days 0, 5, 10, and 15, rapidly frozen in liquid nitrogen, and stored at −80 °C. The experimental methodology followed a completely randomized design, with treatments assigned randomly and each treatment biologically replicated four times.

### Determination of Longan fruit physiological properties

Based on initial weight (day 0) and weights on subsequent sampling days, the percentage of physiological weight loss was calculated as follows: [(fruit Weight on day 0 - fruit weight on sampling day)/fruit Weight on day 0] × 100 = total weight loss (%). A digital refractometer was used to measure total soluble solids (TSS) content, which was then expressed as a percentage. Additionally, the Horwitz method was used to determine titratable acidity (TA). Briefly, 40 mL of distilled water and 10 mL of fruit juice were mixed, and titration carried out using 0.1 N NaOH with phenolphthalein as the indicator.

A colorimeter (Chromameter CR400, Konica Minolta, Japan) was used to assess the color of postharvest longan fruit. Parameters such as L^*^ (lightness), a^*^ (greenness), b^*^ (yellowness), and h (hue angle) values were recorded. The color index (CI) was calculated using the formula: CI = (a^*^ × 1000)/(L^*^ × b^*^). The browning index (BI) was calculated using the formula: BI = 100 × (X − 0.31)/0.17, where X is defined as (a^*^ + 1.75 × L^*^)/(5.645 × L^*^ + a^*^ − 3.012 × b^*^) (Zhou et al., 2022).

Electrolytic leakage and MDA content were measured using previously described procedures (Shan et al., 2016; Yang et al., 2021). Carotenoid, AsA, total phenols, and flavonoid contents were also measured as described previously (Zhou et al., 2022; Wang et al., 2018; Zhang et al., 2015).

ATP, ADP, and AMP measurements were conducted using a Sepex C18 reverse-phase column (250 mm × 4.6 mm) and a high-performance liquid chromatography (HPLC) system, following previously established methods^9^. ATP, ADP, and AMP levels were quantified in µg·g^−1^ based on a new weight basis, using an external standard curve. The EC was calculated using the following formula:

$$\:\text{E}\text{C}=\frac{\text{A}\text{T}\text{P}+1/2\text{A}\text{D}\text{P}}{\text{A}\text{T}\text{P}+\text{A}\text{D}\text{P}+\text{A}\text{M}\text{P}}$$

H^+^-ATPase, Ca^2+^-ATPase, SDH, and CCO activities, proline content, and P5CS, OAT, and PDH activities were determined using assay kits supplied by Suzhou Keming Co. Ltd (Suzhou, China).

### Total RNA extraction, cDNA library construction, and illumina sequencing

Total RNA was extracted from longan fruit tissues using the TRIzol^®^, a plant RNA purification reagent. DNase I (TaKara) was applied to RNA samples to eliminate genomic DNA contamination. RNA sample quality was assessed using an Agilent 2100 Bioanalyzer (Agilent Technologies), and the concentration was measured using a NanoDrop ND-2000. A TruSeqTM RNA sample preparation Kit from Illumina (San Diego, CA) was used to generate the transcriptome library (using 1 µg of total RNA). Polymerase chain reaction (PCR) was carried out using Phusion DNA polymerase (NEB). Subsequently, the RNA-seq Libraries underwent paired-end sequencing on the Illumina NovaSeq 6000 platform (Illumina, San Diego, CA, USA). The resulting raw data were submitted to the National Center for Biotechnology Information (NCBI) database with the accession number PRJNA1049125 (https://www.ncbi.nlm.nih.gov/bioproject/PRJNA1049125).

### Quality control, read mapping, and enrichment analysis of the differentially expressed genes (DEGs)

Raw paired-end reads were trimmed using fastp with default settings (https://github.com/OpenGene/fastp). HISAT2 software (http://ccb.jhu.edu/software/hisat2/index.shtml) was used in orientation mode to independently align clean reads to the reference genomes. To assemble mapped reads for each sample, we employed StringTie (https://ccb.jhu.edu/software/stringtie/) using a reference-based approach. Gene expression levels and differentially expressed genes (DEGs) across groups were assessed using the transcripts per million (TPM) read technique. Gene abundance was quantified with RSEM (http://deweylab.biostat.wisc.edu/rsem), and DEGs were calculated using DEGSeq. The adjusted *p*-values (*P*-adjust) were calculated using the Benjamini-Hochberg (BH) method for controlling the false discovery rate (FDR). DEGs with |log2 (foldchange)| ≥ 1 and *P*-adjust ≤ 0.001 were considered statistically significant.

For functional enrichment analysis, we used the Gene Ontology (GO) and Kyoto Encyclopedia of Genes and Genomes (KEGG) databases. DEGs were analyzed for significant enrichment in GO terms and metabolic pathways at a *P*-adjust ≤ 0.05 level relative to the entire transcriptome background. KOBAS (http://kobas.cbi.pku.edu.cn/home.do) and Goatools (https://github.com/tanghaibao/Goatools) were employed for GO functional enrichment and KEGG pathway analyses.

### Quantitative real-time (qRT)PCR analysis

We employed quantitative real-time (qRT)-PCR to validate the expression patterns of nine genes selected from the transcriptome analysis data. Primer Premier version 5.0 was used to design the primers (Table S1). Using the *Actin* gene as a reference gene, we applied the delta-delta Ct (2^−ΔΔCt^) method, to determine the relative expression levels of the differentially expressed genes (DEGs) in the various samples.

### Statistical analysis

We employed SPSS software (version 19.0) to independently analyze the data from each group. Statistical significance was assessed using Tukey’s test with a significance level set at *P* < 0.05, and different letters were used to denote significant differences.

## Results

### Specific concentrations of CTS improved the storage quality of longan fruit during post-harvest

**Fig. 1:**
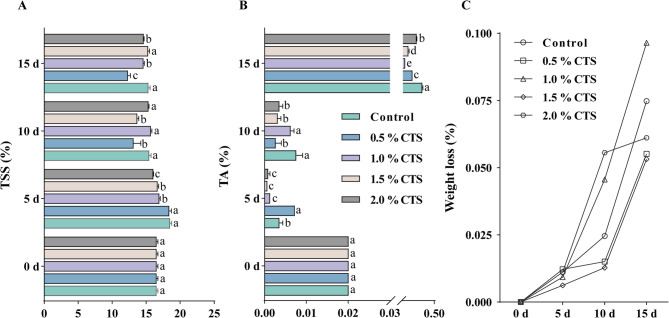
The effects of different concentrations of CTS treatment on the SSC content (A), TA content (B), and weight loss rate (C) of postharvest longan. Bars with a different letter within a sampling date are significantly different (*P* < 0.05).

CTS is widely utilized in the post-harvest preservation of fruit and vegetables to maintain their quality. While CTS is applied in the post-harvest preservation of longan, the optimal concentration for its effective use remains undetermined. To determine the optimal CTS concentration for post-harvest preservation, we initially assessed the effects of 0.5%, 1.0%, 1.5%, and 2.0% CTS coatings on post-harvest longan fruit. This evaluation involved measuring SSC, TA levels and Weight loss rates. Compared with the control, longan fruit TSS declined with 0.5, 1.0, 1.5, and 2.0% CTS. Specifically, 0.5% CTS significantly decreased TSS content by 14.98 and 19.57% after 10 and 15 days of storage, respectively (Fig. 1A). During the early and middle stages of storage, the TA content of postharvest longan fruit remained low. However, by the 15th day of storage, the 0.5%, 1.0%, 1.5%, and 2.0% CTS treatments significantly reduced the TA content by 43.99%, 77.19%, 62.53%, and 25.37%, respectively, with the 1.0% concentration maintaining the lowest level (Fig. 1B). Postharvest longan fruit Weight loss varied with different CTS treatment concentrations, with 1.5% CTS having the lowest weight loss during the entire postharvest storage period (Fig. 1C). We hypothesize that CTS reduces the fruit’s acidity by inhibiting the growth of post-harvest microbial populations in longan fruit through its potent antibacterial properties, thereby decreasing the production of acidic metabolic products by these microbes.

**Fig. 2:**
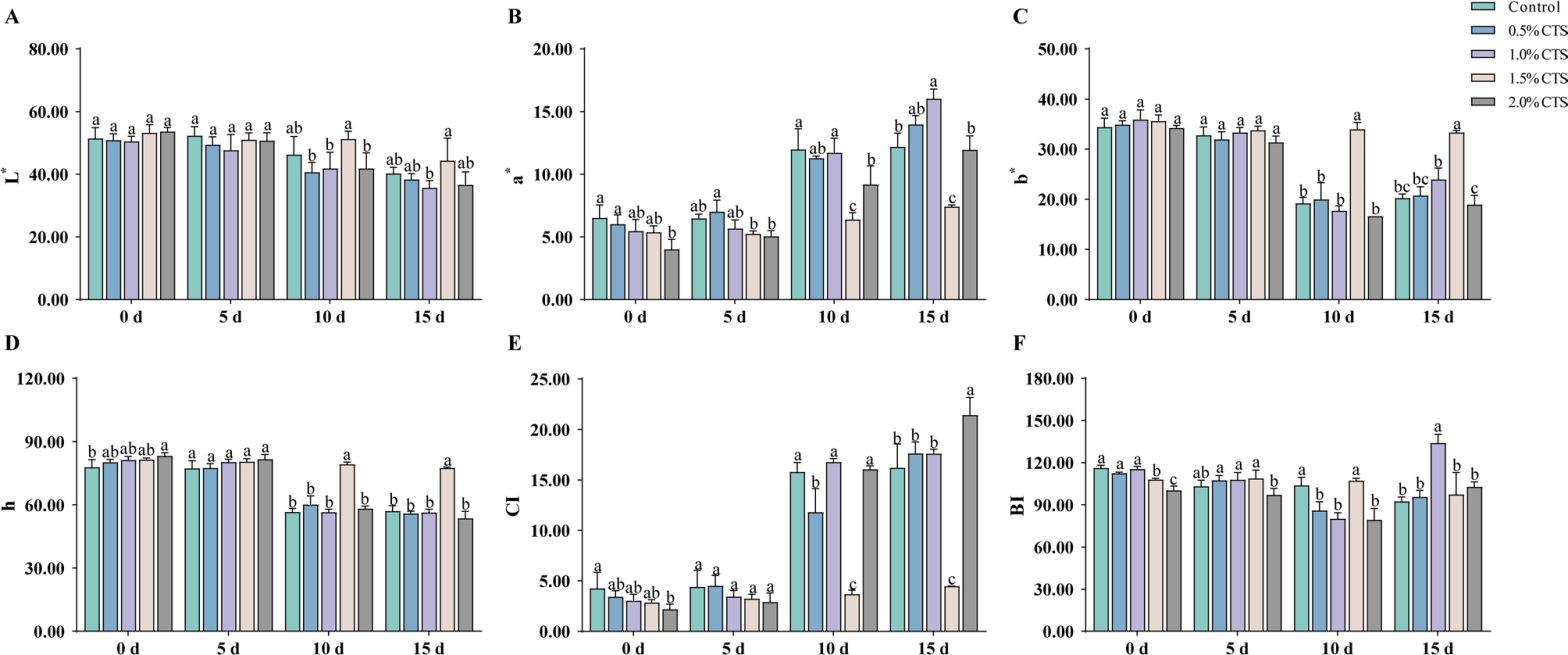
The effects of different concentrations of CTS treatment on the color changes of the peel of postharvest longan. L* (lightness) value (A), a* (greenness) (positive values) (B), b* (yellowness) (C), h (hue angle) (positive values) (D), color index (positive values) (E), and browning index (F). Error bars represent SD (*n* = 4). Bars with a different letter within a sampling date are significantly different (*P* < 0.05).

Fruit peel color is a crucial factor influencing the commercial value of longan and serves as an effective indicator for assessing post-harvest storage quality. We assessed the impact of various concentrations of CTS coatings on the color characteristics of post-harvest longan fruit peel. This evaluation included measurements of L^*^ values (lightness), a^*^ values (red-green spectrum), b^*^ values (yellow-blue spectrum), h^*^ values (hue angle), color index, and brightness index. While different CTS treatments altered longan fruit peel L^*^ values to varying degrees, there were no significant differences (Fig. 2A). 1.0% CTS significantly increased peel a^*^ values by 31.47% after 15 days of storage, while 1.5% CTS significantly decreased it by 46.59 and 39.21% on 10 d and 15 d storage, respectively (Fig. 2B). Compared to the control, 1.5% CTS significantly increased peel b^*^ and h^*^ values by 77.14 and 64.46% after 10 days of storage and by 40.16% and 35.78% after 15 days of storage, respectively (Fig. 2C and D). The 1.5% CTS resulted in significant decreases of 76.68 and 72.37% in the CI after 10 days and 15 days of storage (Fig. 2E). The 1.0% CTS significantly reduced the BI by 22.87% after 10 days of storage and significantly increased it by 45.05% after 15 days of storage, compared to the control (Fig. 2F).

**Fig. 3:**
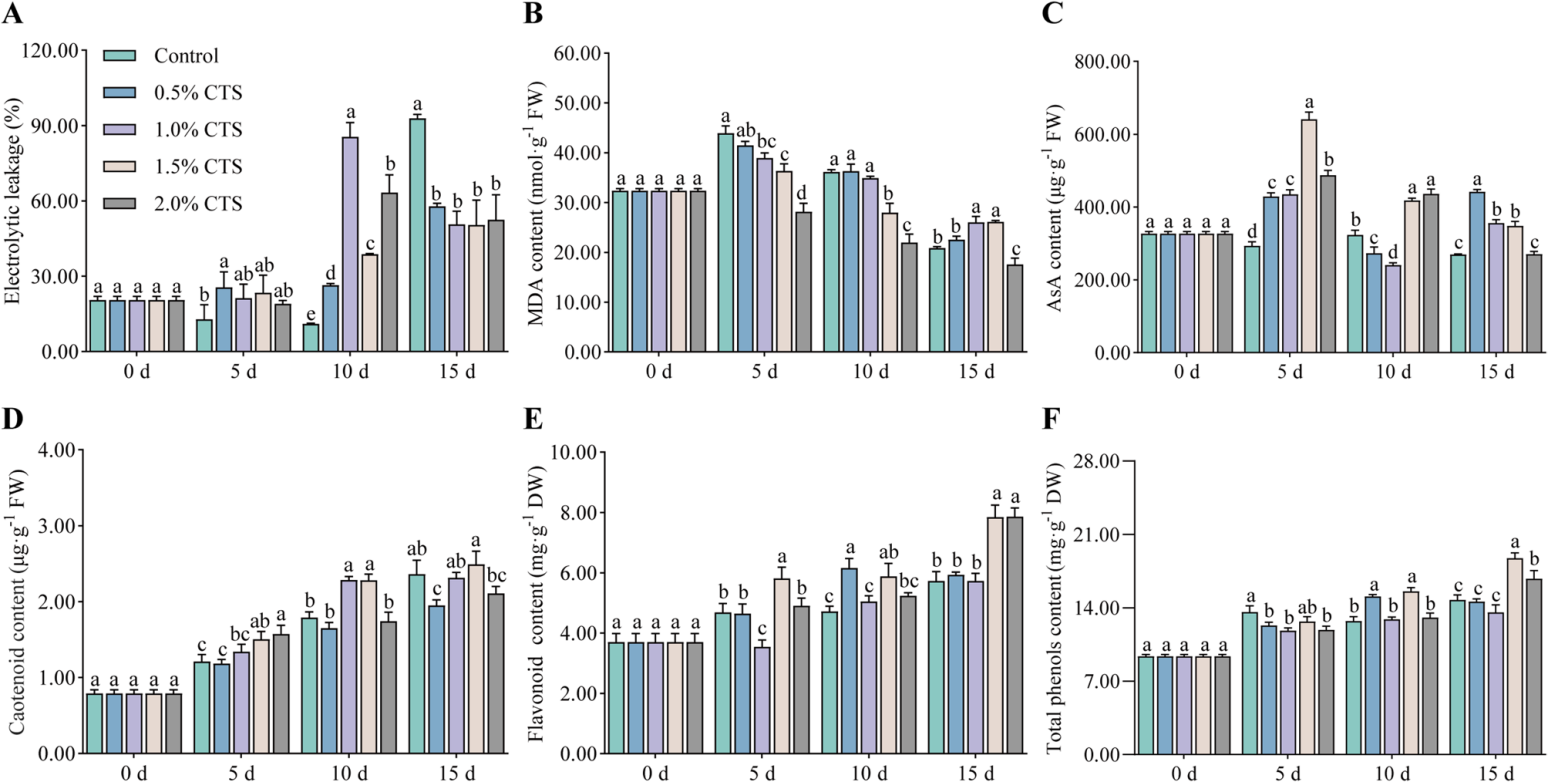
Effects of chitosan treatment concentration on postharvest longan quality. indicators of membrane integrity (A, B) and antioxidant levels (C-F). (A) electrolytic leakage and (B) MDA content indicating cell membrane integrity. (C) AsA, (D) carotenoid, (E) total phenol, and (F) flavonoid contents representing antioxidant levels.

The loss of cell membrane integrity is a primary indicator of post-harvest fruit decay. Accordingly, we will evaluate the effectiveness of CTS in preserving the cell membrane integrity of post-harvest longan fruit, specifically through measurements of electrical conductivity and MDA content. These assessments will yield insights into the extent of membrane damage and lipid peroxidation, which are critical factors in determining the health and longevity of stored fruit. In the early storage period (5 days), 0.5% CTS significantly increased longan fruit peel electrolytic leakage. In the mid storage period, 0.5, 1.0, 1.5, and 2.0% CTS significantly increased peel electrolytic leakage (Fig. 3A). Longan fruit peel MDA content decreased with increasing storage time. Compared to the control, 1.0% CTS significantly reduced peel MDA content at 5 days storage and significantly increased it at 15 days storage. The 1.5% CTS resulted in significant reductions of 17.21 and 22.63% in peel MDA content at 5 and 10 days of storage, respectively, and a significant increase of 25.27% at 15 days of storage. The 2.0% CTS significantly reduced peel MDA content by 15.84–39.18% during the entire storage period (Fig. 3B).

Antioxidants in fruit can significantly reduce the damage caused by reactive oxygen species to cells, thereby helping to maintain the quality of post-harvest fruit to some extent. Subsequently, we will evaluate the effect of CTS treatment on the antioxidant levels in post-harvest longan fruit. This assessment will involve measuring the content of four key antioxidants: AsA, carotenoid, flavonoids and total phenolics. At 10 days storage, the 0.5 and 1.0% CTS significantly reduced AsA content by 15.44 and 25.57%, respectively. The 1.5% CTS significantly increased AsA content during the entire storage period, while 2.0% CTS significantly increased it by 65.99% after 5 days, and 35.05% after 10 days of storage (Fig. 3C). Carotenoid contents increased with increasing storage time. Compared with the control, 0.5% CTS significantly reduced longan fruit carotenoid content, while 1.0% CTS significantly increased it by 27.59% after 10 days storage. The 1.5% and 2.0% CTS significantly increased carotenoid content after 5 and 10 days of storage (Fig. 3D). Compared with the control, 0.5% CTS significantly increased longan fruit flavonoid content after 10 days of storage, and 1.0% CTS significantly reduced it by 24.40% after 5 days. The 1.5% CTS treatment significantly increased flavonoid content during the entire storage period, and 2.0% CTS significantly increased it by 37.23% after 15 days of storage (Fig. 3E). At 5 days storage, compared with the control, 0.5, 1.0, and 2.0% CTS significantly reduced longan fruit total phenol content by 9.32, 13.11, and 12.46%, respectively. At ten days storage, 0.5 and 1.5% CTS significantly increased the total phenol content (Fig. 3F).

**Fig. 4:**
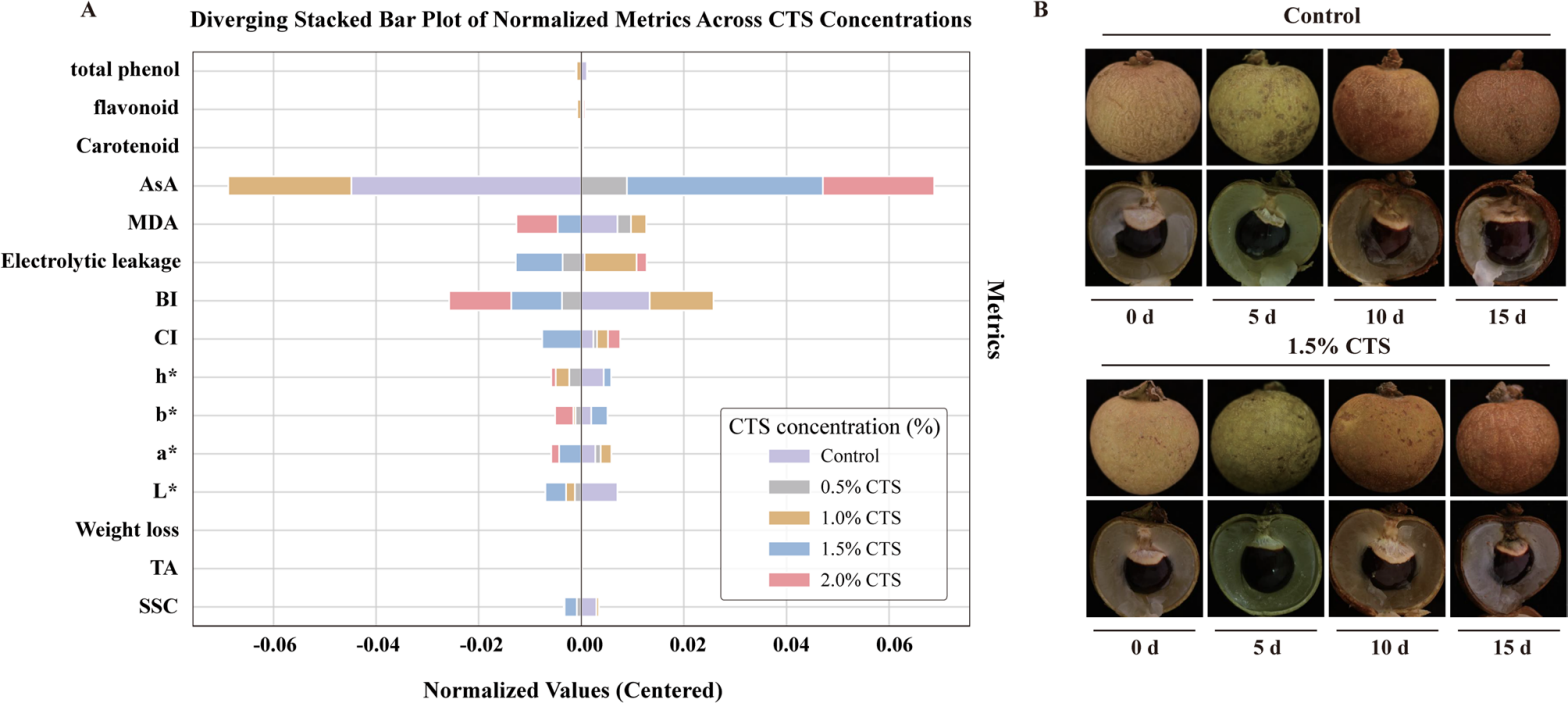
Different concentrations of chitosan preservation effects and phenotypic analysis. (a) Weight analysis of different indices of postharvest longan preservation affected by various concentrations of chitosan. (b) Phenotypic analysis of the extension of longan shelf Life using 1.5% chitosan.

Based on the evaluation and analysis of various CTS concentrations, we have determined that a 1.5% CTS treatment enhances the storage quality of longan, maintaining cell membrane integrity and reducing damage caused by reactive oxygen species, compared to concentrations of 0.5%, 1.0%, and 2.0% (Fig. 4A). Consequently, we selected longan fruit treated with 1.5% CTS at 0 and 15 days of storage for transcriptome analysis (Fig. 4B). This investigative approach aims to identify gene expression changes and regulatory pathways activated by CTS treatment that contribute to its preservative effects.

### Transcriptome analysis reveal the regulatory mechanisms of 1.5% CTS treatment enhances the quality of post-harvest longan fruit

**Fig. 5:**
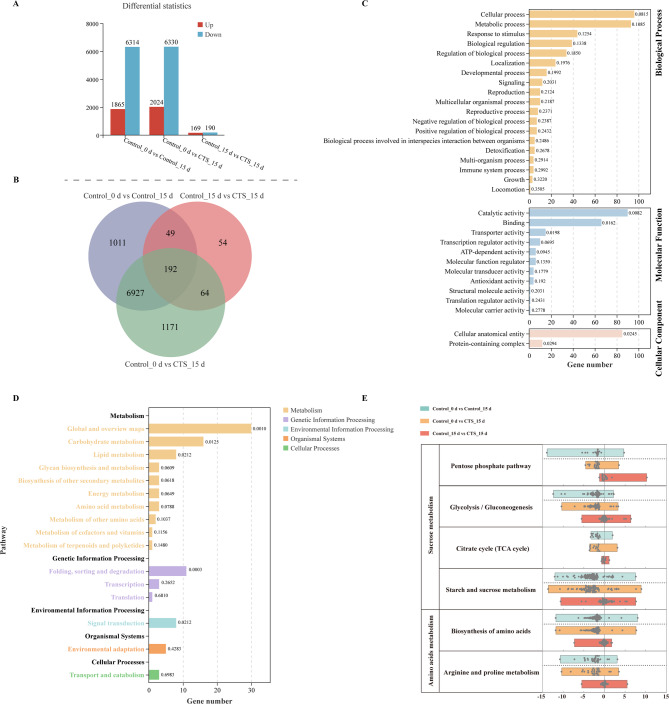
Analysis of gene expression during ripening and modulated by CTS in longan fruit. Analysis of differentially expressed genes (DEGs) analysis (A). Venn diagrams analysis (B). GO analysis (C). KEGG analysis (D). DEGs with the annotated functions in sucrose metabolism and amino acids metabolism (E). In panel (E), each dot within the bars represents an individual gene participating in the respective metabolic pathway, and the x-axis position of each dot corresponds to the Log_2_FC (log_2_fold change) value of that gene.

Longan fruit gene expression profiles were analyzed after treatment with 1.5% CTS using transcriptome sequencing. The average mapping rate for these reads was 73.00% (Table S2). Correlation coefficients among pairs of biological replicate samples indicated a high level of consistency in gene expression within each replicate (Figure S1). We next conducted a differential expression analysis to identify specific gene expression alterations. This comparison was made between differentially expressed genes (DEGs) in control and CTS-treated groups at each storage time point. In the Control_0 d vs. Control_15 d comparison, 1865 genes were upregulated, and 6314 were downregulated. For the Control_0 d vs. CTS_15 d comparison, 2024 genes were upregulated, and 6330 were downregulated. Moreover, the Control_15 d vs. CTS_15 d comparison revealed 169 upregulated and 190 downregulated genes (Fig. 5A). The majority of DEGs were shared between control and CTS-treated fruit at each evaluated time point, with 192 DEGs identified in all three comparisons (Fig. 5B).

To determine the function of these differentially expressed genes, we conducted a GO enrichment and KEGG pathway analysis to determine the function of the 192 common DEGs. The predominant terms included ‘cellular process,’ ‘metabolic process,’ and ‘response to stimulus’ in biological processes; ‘catalytic activity,’ ‘binding,’ and ‘transporter activity’ in molecular functions; and ‘cellular anatomical entity’ and ‘protein-containing complex’ in cellular components. Moreover, ‘ATP-dependent activity’ and ‘antioxidant activity’ were notably enriched, indicating their association with antioxidant responses and energy metabolism (Fig. 5C and Table S3). Subsequently, we analyzed the 192 DEGs using the KEGG pathway database. The principal metabolic pathways affected were carbohydrate metabolism, lipid metabolism, and glycan biosynthesis and metabolism (Fig. 5D), as depicted in Table S4. Furthermore, among the 192 common DEGs, other prominently featured pathways encompassed folding, sorting and degradation, signal transduction, environmental adaptation, transport, and catabolism.

Next, further comprehensive analysis delineated the impacts of CTS treatment (CTS) on various metabolic and regulatory pathways in stored longan fruit. We found that CTS treatment (CTS) induces alterations in sucrose and amino acid metabolism in postharvest longan fruit. Differential gene expression was noted in the pentose phosphate, glycolysis/gluconeogenesis, citrate cycle (TCA cycle), and starch and sucrose metabolism pathways. In the Control_15 d versus CTS_15 d comparison, 8, 24, 6, and 39 genes were upregulated, while 13, 31, 9, and 40 genes were downregulated in these respective pathways (Fig. 5E and Table S5). We found that CTS treatment also resulted in differential gene expression in the amino acids biosynthesis and arginine and proline metabolism pathways. In the Control_15 d versus CTS_15 d comparison group, forty-three genes were upregulated, while fifty-seven genes were downregulated. Specifically, in the arginine and proline metabolism pathway, seventeen genes showed upregulation and thirteen genes showed downregulation (Fig. 5E and Table S5). These findings suggest that CTS treatment can modulate gene expression related to sucrose and amino acid metabolism, thereby influencing the senescence of longan fruit postharvest.

### CTS treatment maintain fruit quality of post-harvest longan by regulating energy and proline metabolism

**Fig. 6:**
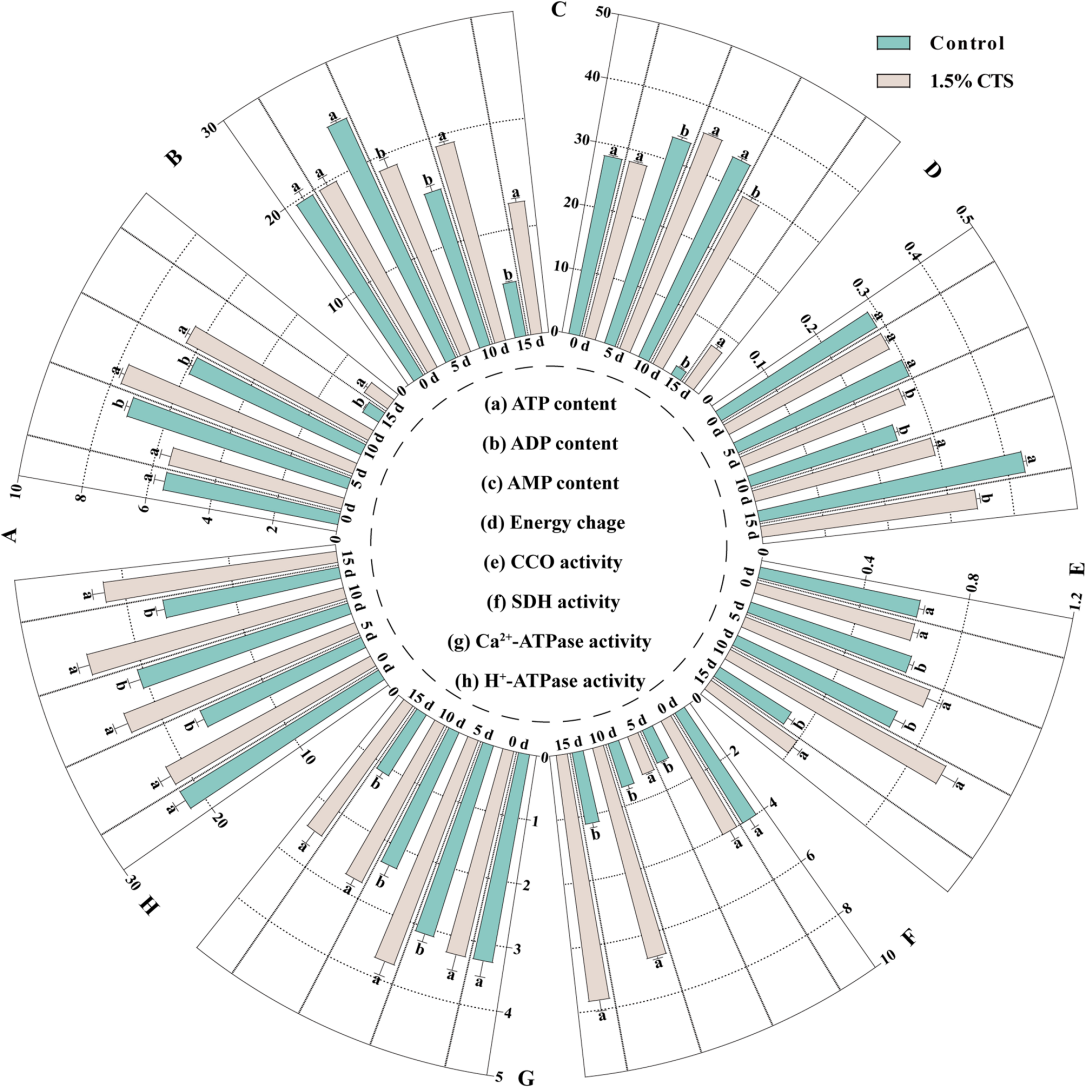
Effects of 1.5% chitosan treatment on the energy levels and activities of enzymes related to energy metabolism in postharvest longan fruit. Energy levels are assessed by ATP (a), ADP (b), AMP (c) content, and energy change (d). Enzymes related to energy metabolism include CCO (E), SDH (F), Ca^2+^-ATPase (G) and H^+^-ATPase (H). Error bars represent SD (*n* = 4). Bars with different letters within a sampling date indicate significant differences (*P* < 0.05).

**Fig. 7:**
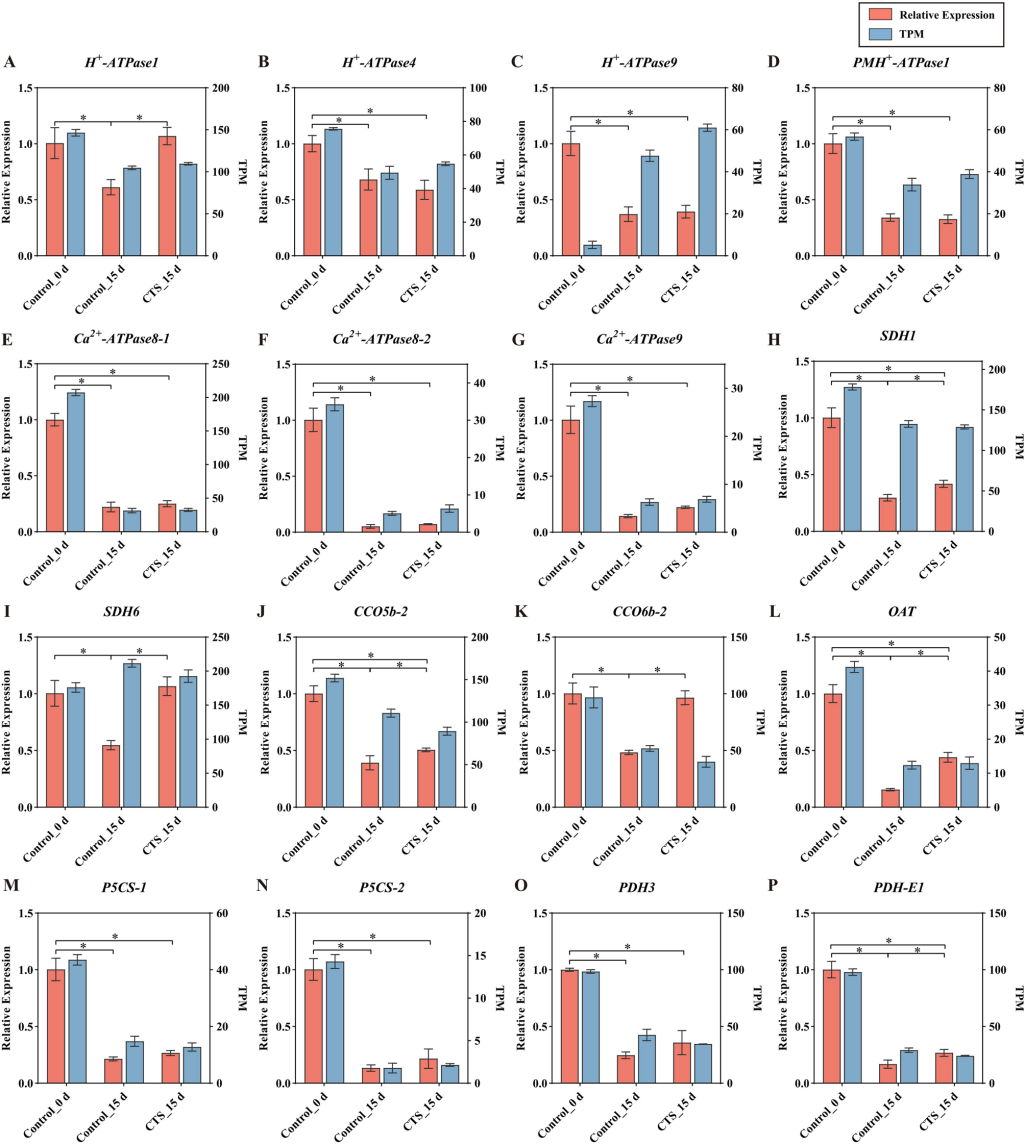
Expression levels of 16 selected DEGs related to energy metabolism and amino acid/proline metabolism. *H*^*+*^-*ATPase1*(A), *H*^*+*^*-ATPase4*(B), *H*^*+*^*-ATPase9*(C), *PMH*^*+*^*-ATPase4*(D), *Ca*^*2+*^*-ATPase8-1*(E), *Ca*^*2+*^*-ATPase8-2*(F), *Ca*^*2+*^*-ATPase9*(G), *SDH1*(H), *SDH6*(I), *CCO5b-2*(J), *CCO6b-2*(K), *OAT*(L), *P5CS-1*(M), *P5CS-2*(N), *PDH3*(O), and *PDH-E1*(P). Data are presented as mean ± standard deviation (*n* = 3). Pairwise analysis of variance (ANOVA) with post-hoc test was performed to compare each CTS treatment group to the untreated control group (Control). Asterisks (*) indicate significant differences at *P* < 0.05.

To elucidate the regulatory effects of CTS on sucrose and amino acid metabolism in post-harvest longan, we measured related substances, enzyme activities, and gene expression changes associated with energy and proline metabolism. We assessed cellular energy levels in post-harvest longan by measuring ATP, ADP, AMP, and EC levels, as well as the activities of H^+^-ATPase, Ca^2+^-ATPase, SDH, and CCO. Our findings indicate that ATP content in longan fruit initially increased and then decreased, reaching its lowest on the 15th day of storage (Fig. 6A). Compared to the control, 1.5% CTS treatment significantly elevated ATP content by 7.13 to 49.10%. ADP content significantly decreased at 5 days and increased at both 10 and 15 days of storage (Fig. 6B). AMP content mirrored ATP content changes (Fig. 6C). EC levels remained relatively high until the 15th day, with 1.5% CTS notably reducing EC levels at 5 and 15 days of storage, and increasing it by 20.81% at 10 days (Fig. 6D). We also evaluated mitochondrial function in post-harvest longan cells by measuring SDH and CCO activities, which significantly increased with CTS treatment (Fig. 6E-F). Further analyses of key enzymes activity in energy metabolism revealed that 1.5% CTS maintained high H^+^-ATPase levels throughout the storage period (Fig. 6H), while Ca^2+^-ATPase levels gradually decreased (Fig. 6G). Compared to the control, 1.5% CTS significantly boosted both H^+^-ATPase and Ca^2+^-ATPase activities during the storage. Additionally, gene expression analysis using qRT-PCR and RNA-seq indicated consistent expression patterns, validating that RNA-seq reliably reflects gene expression profiles similar to qRT-PCR results. In terms of energy metabolism, genes like *H*^*+*^*-ATPase1*, *SDH1*, *SDH6*, *CCO5b-2*, and *CCO6b-2* showed increased expression following CTS treatment on the 15th day, inversely correlating with fruit senescence (Fig. 7A-K).

**Fig. 8:**
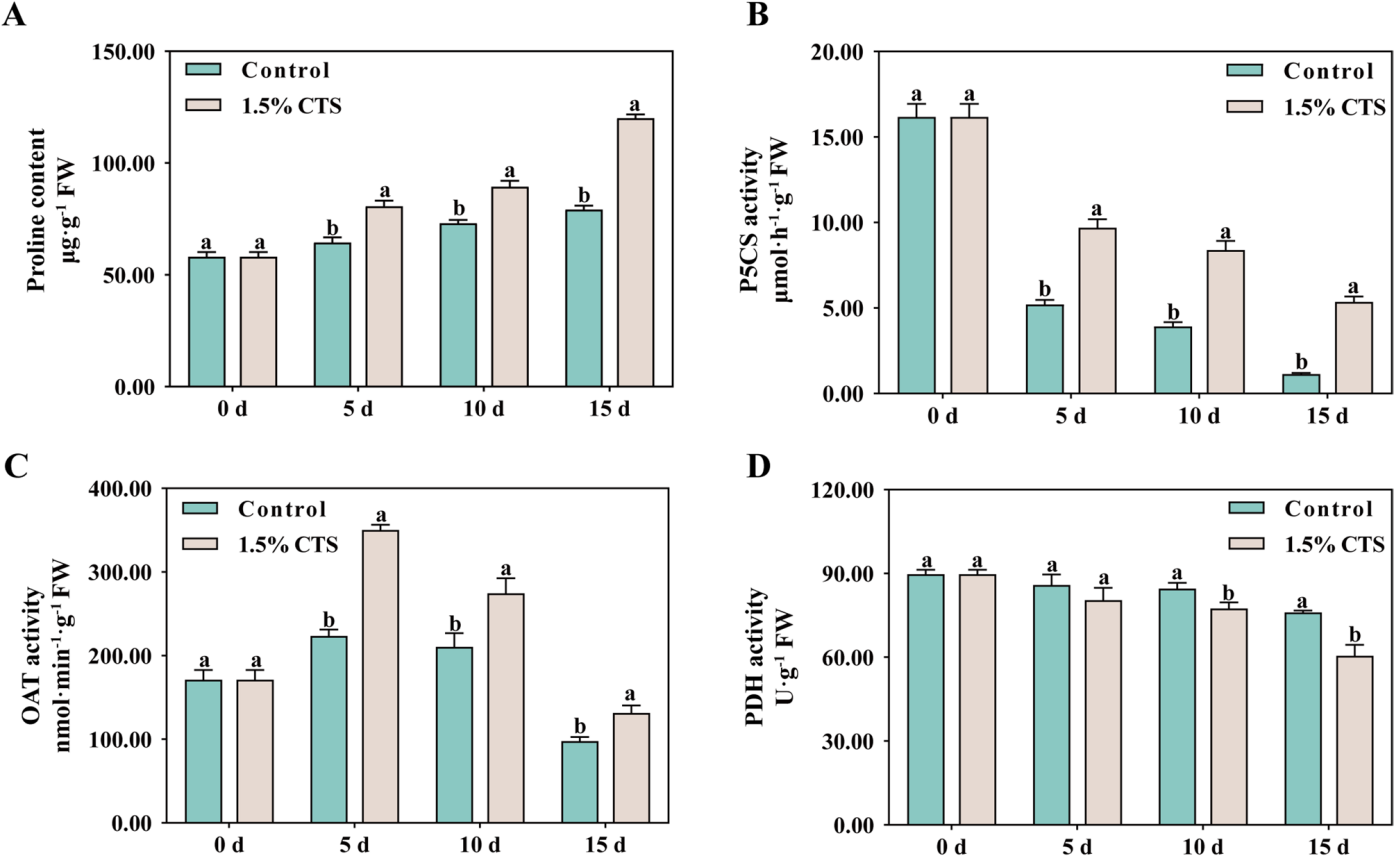
Proline content (A), P5CS (B), OAT (C), and PDH (D) activities in different concentrations of CTS treatment in longan fruit during storage. Error bars represent SD (*n* = 4). Bars with a different letter within a sampling date are significantly different (*P* < 0.05).

We further explored the effects of CTS treatment on proline metabolism in post-harvest longan fruit. Results showed that proline content in the fruit gradually increased with extended storage time (Fig. 8A). Specifically, 1.5% CTS treatment significantly elevated proline content throughout the entire storage period. Meanwhile, P5CS activity in longan fruit decreased progressively with increasing storage time (Fig. 8B); however, 1.5% CTS treatment markedly enhanced P5CS activity, with increases ranging from 86.38 to 371.54% over the storage period. OAT activity initially rose and then declined as storage time extended (Fig. 8C), yet 1.5% CTS consistently boosted OAT activity throughout the storage period. In comparison with the control, 1.5% CTS treatment significantly reduced PDH activity in longan fruit at 10 and 15 days of storage (Fig. 8D). Furthermore, analysis of proline metabolism revealed that CTS treatment increased the expression levels of the OAT and PDH-E1 genes, underscoring their significant roles in regulating postharvest fruit senescence (Fig. 7L-P).

## Discussion

The effectiveness of chitosan (CTS), including its antimicrobial properties and ability to form a protective film, is concentration-dependent^8,11^. At low concentrations, the oxygen barrier effect is diminished, resulting in increased oxygen content and respiration rates, which hasten nutrient depletion. In contrast, high concentrations produce a thicker film that restricts oxygen flow, leading to anaerobic respiration and accelerated decay^13,28^. Due to variations in fruit development among different varieties within the same type of fruit and vegetables, the preservation effect of chitosan coatings at the same concentration can vary significantly^1^. To further analyze the role of chitosan in the post-harvest preservation of longan fruit, it is essential to identify the optimal chitosan concentration suitable for preserving specific longan varieties. Our research assessed the impact of various CTS concentrations on the physiological characteristics, quality, and storage capabilities of longan fruit. The results revealed that a 1.5% CTS concentration most effectively reduces post-harvest respiration rates compared to concentrations of 0.5%, 1%, and 2%, thereby preserving nutrients such as TSS and AsA^7^. Additionally, this concentration curtails the increase in cell membrane permeability caused by lipid peroxidation due to reactive oxygen species and pathogens, thereby preserving membrane integrity^28^. The 1.5% CTS treatment also effectively inhibits the production of acidic metabolic products by pathogens, preventing acidification and sour rot in longan fruit, and maintains higher levels of carotenoids, ascorbic acid, total phenols, and flavonoids, thereby enhancing antioxidant capacity and overall storage quality^29^. Consequently, a 1.5% CTS concentration is effective in maintaining the quality of post-harvest longan fruit and extending their shelf life.

Chitosan has been applied as a new eco-friendly preservative in the field of fruit and vegetable preservation, effectively replacing most chemical preservatives due to its excellent preservative performance^30,31^. However, critical research on the preservation mechanism of chitosan remains limited. Energy deficit was considered as the primary reason for the loss of commercial value in tropical fruit during post-harvest storage^3,11,19^, Maintaining a high energy level in fruit is therefore considered a key measure to extend their shelf life^27,32^. Through KEGG and GO analysis of transcriptomic changes in longan fruit after chitosan treatment, we found that chitosan not only affects the expression of disease resistance-related genes but also influences the expression of genes related to energy metabolism in longan fruit (Fig. 5). We further measured the energy levels and the activity of energy metabolism-related enzymes in longan fruit after chitosan treatment (Fig. 6). A 1.5% CTS treatment effectively maintained high ATP and ADP levels, along with low AMP levels, during the mid-storage period, resulting in high EC levels. This provided sufficient energy for cellular repair processes and enhanced the storage capability of longan fruit. Our research indicates that a 1.5% CTS treatment increased the activity of H^+^-ATPase and Ca^2+^-ATPase in longan fruit during storage, particularly elevating the expression of *H*^*+*^*-ATPase 1* in the later stages, thereby supporting the energy required for normal physiological functions and further enhancing storage capability.

**Fig. 9:**
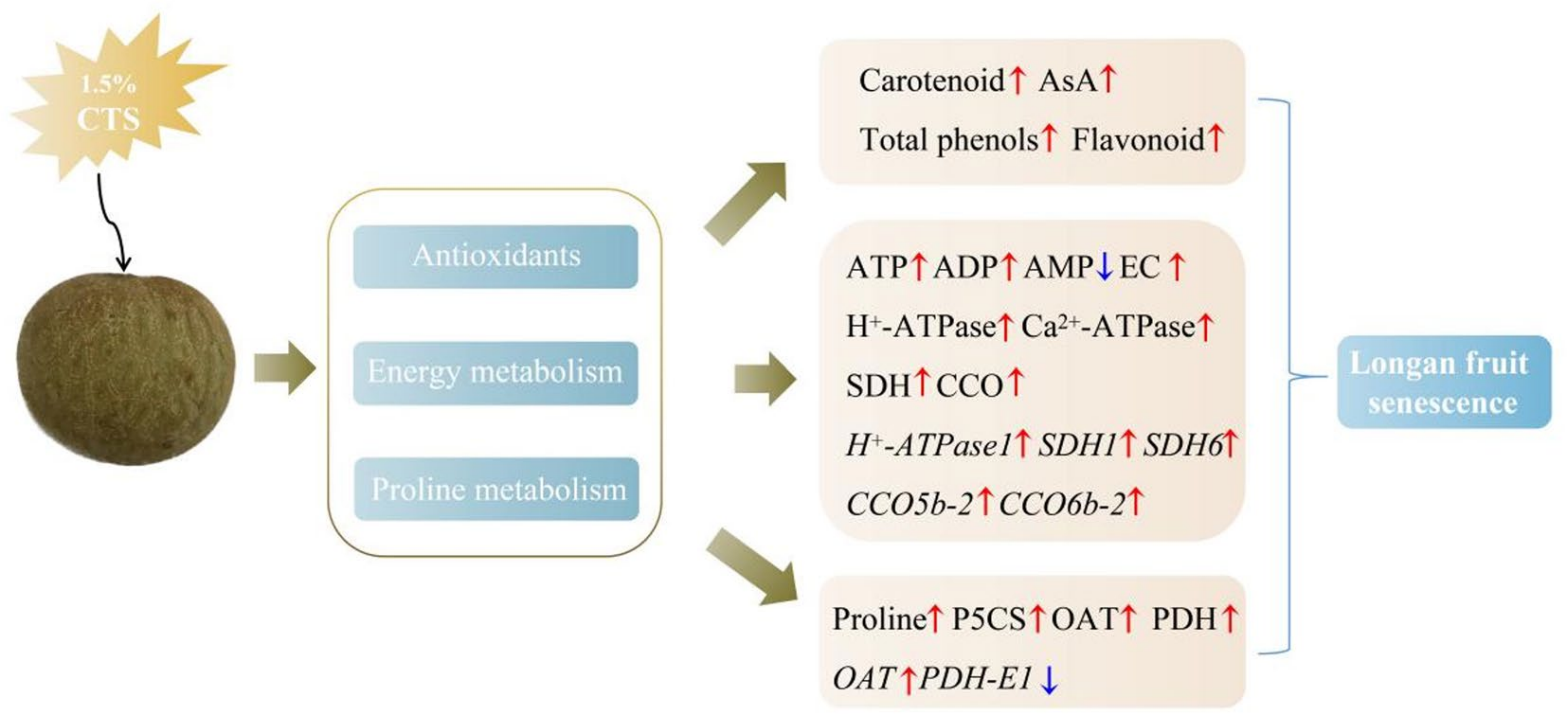
The model pattern of longan fruit senescence as mediated by CTS. Red arrows indicate up-regulation, while blue arrows indicate down-regulation.

Previous studies have suggested that microbial infection is a primary cause of energy deficit in post-harvest fruit^31,33–35^. Research has shown that in longan fruit infected with *Phomopsis longanae Chi*, energy levels decrease, as evidenced by a reduction in ATP and ADP levels, an increase in AMP levels, and a decrease in energy charge. These alterations in energy metabolism are closely associated with fruit browning and disease progression, underscoring the importance of maintaining high energy levels to extend the storage period of fruit^36^. Therefore, reducing microbial infection is beneficial for maintaining high energy levels in fruit, effectively extending their post-harvest shelf life. We believe that chitosan, as an effective antimicrobial coating material, can reduce microbial infection and proliferation in post-harvest fruit, thereby minimizing energy dissipation and helping to maintain high energy levels^37,38^. This conservation of energy and moisture effectively allocates resources for other physiological metabolic functions, ultimately extending the post-harvest shelf life of longan fruit (Fig. 9).

As a crucial osmotic regulator, proline plays a key role in increasing osmotic pressure, stabilizing cell membranes and subcellular structures, and mitigating oxidative stress. This contributes to preserving membrane integrity and improving the storability of fruit. The regulation of proline levels in fruit and vegetables involves the enzymes ornithine aminotransferase (OAT), P5CS, and PDH, where OAT and P5CS are responsible for proline synthesis, and PDH for its degradation, ensuring a balance between synthesis and breakdown^39^. Treatment with glycine betaine notably increased P5CS and OAT activities in peaches, simultaneously decreasing PDH activity, thereby raising proline concentrations and enhancing cold tolerance. Furthermore, the application of exogenous ethylene has been shown to promote proline accumulation in fruit peels by boosting P5CS and OAT activities, upregulating *P5CS1* and *OAT1* gene expressions, and reducing both the activity and expression of *PDH1*, thus aiding in stress resistance^40^. Similarly, treating postharvest loquat with CaCl_2_ during cold storage amplified P5CS and OAT activities^7^. Our research reveals that CTS treatment elevates proline levels in longan fruit by enhancing OAT and P5CS activities and OAT expression levels, while diminishing PDH activity, consequently fostering improved storability.

## Supplementary Information

Below is the link to the electronic supplementary material.

Supplementary Material 1

Supplementary Material 2

Supplementary Material 3

Supplementary Material 4

Supplementary Material 5

Supplementary Material 6

Supplementary Material 7

## Data Availability

The datasets generated and analyzed during the current study are available in the NCBI repository under the accession number PRJNA1049125.
