# Supplementary material for: Enhancement of postharvest longan fruit quality through chitosan (CTS)-induced modulation of energy and proline metabolism
Source: Sci Rep. 2025 Sep 29;15:33408. doi: 10.1038/s41598-025-18761-w (PMC12479863; doi:10.1038/s41598-025-18761-w)
Supplement: Supplementary file 1 — Supplementary Material 1 [file 41598_2025_18761_MOESM1_ESM.docx]

| Gene name | Gene ID | Primer sequences (5’-3’) | Product size (bp) |
| --- | --- | --- | --- |
| *H^+^-ATPase1* | Dil.07g011500.1 | TCACTGCCAAGCCCGTCGTTT | 318 |
|  |  | GCCTTTGTCTGCCGCCTAATT |  |
| *H^+^-ATPase4* | Dil.01g008430.1 | GGACCCAACAAACTGGAAGAAAA | 384 |
|  |  | AAGGATCACCCTCAAGAAGACGA |  |
| *H^+^-ATPase9* | Dil.02g027380.1 | TGGCAGGAACGAGCGTAGAAA | 252 |
|  |  | GCAAAGCCCAAAATGGCATAA |  |
| *PMH^+^-ATPase1* | Dil.14g000550.1 | AACCCAACCGACAAGCGAAC | 456 |
|  |  | ATCAACCGGCAAAGCAACAA |  |
| Ca^2+^-ATPase8-1 | Dil.01g012340.1 | ATCCGCTAATCCACACTCCGAC | 419 |
|  |  | TCATCATCACCATTGATCCCCT |  |
| Ca^2+^-ATPase8-2 | Dil.01g012290.1 | GCCGCTAATACACACTCCGACGA | 372 |
|  |  | CTGACAGCCCTTTAACCCCCCCA |  |
| Ca^2+^-ATPase9 | Dil.03g018530.1 | AATTACACCCCCTACTCCTAGCG | 346 |
|  |  | CATACCATCCTTCTTTCACACCC |  |
| SDH1 | Dil.06g004000.1 | ATTCCCGACTCGTTCGCACACT | 426 |
|  |  | ACTCCCTGGCAGCTACCATCAC |  |
| SDH6 | Dil.13g015510.1 | GCCACTTCCCTCTTGGTCCTCC | 321 |
|  |  | CTGCCCCTCAGACTTCCTTTCC |  |
| *CCO5b-2* | Dil.03g008910.1 | CTTCCCTGTTGGTCCTTTCGG | 152 |
|  |  | GCATTCATGTGGCTTGCCTTT |  |
| *CCO6b-2* | Dil.01g017450.1 | CCAACAACCAACCAAACCAGAC | 153 |
|  |  | GCTCATTCCACCTATCAACCCA |  |
| *OAT* | Dil.07g012650.1 | GAAGTTGGCTAGGAAGTGGGG | 238 |
|  |  | GAAATGCAGCTATGCGGTCTC |  |
| *P5CS-1* | Dil.14g004570.1 | TGCGGCAGACAATATAATAAAGGT | 298 |
|  |  | CAAGGCCAAACGAGATACGAGAGA |  |
| *P5CS-2* | Dil.04g025710.1 | GGGGTAAGGAGGCAAAACGA | 402 |
|  |  | CAGCCCGAAGGTCAACAATG |  |
| *PDH3* | Dil.03g002350.1 | TGCCCTCTCTTTCACCAACAAT | 173 |
|  |  | CTCCATCCCCCTTCAAAATCTT |  |
| *PDH-E1* | Dil.10g016080.1 | CCATCTCTACGACGGACAGGAA | 264 |
|  |  | AAGCCGCAACCCAAGGGAATCT |  |
