# Supplementary figures and images for: Enhancement of postharvest longan fruit quality through chitosan (CTS)-induced modulation of energy and proline metabolism
Source: Sci Rep. 2025 Sep 29;15:33408. doi: 10.1038/s41598-025-18761-w (PMC12479863; doi:10.1038/s41598-025-18761-w)

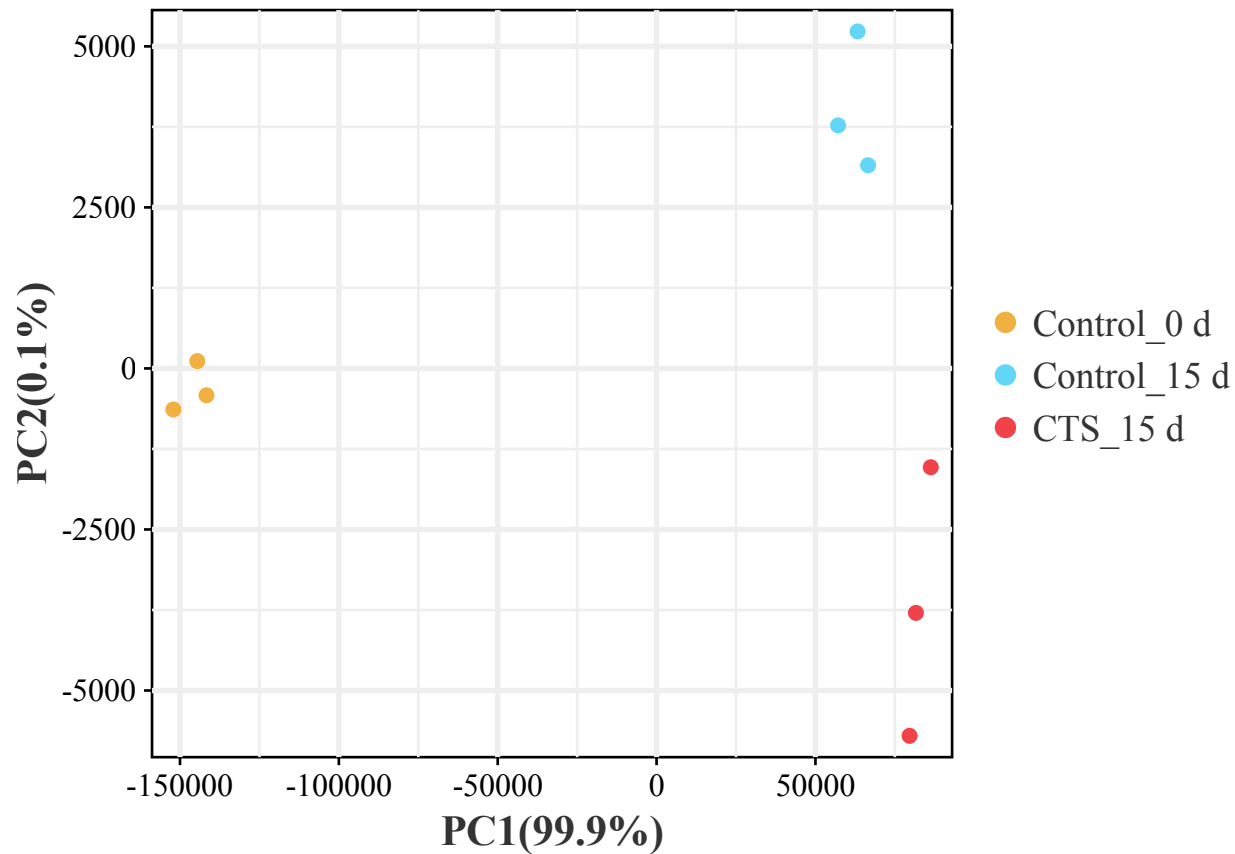

Supplement: Supplementary file 6 — Supplementary Material 6 [file 41598_2025_18761_MOESM6_ESM.pdf]
