# Supplementary material for: Enhancement of postharvest longan fruit quality through chitosan (CTS)-induced modulation of energy and proline metabolism
Source: Sci Rep. 2025 Sep 29;15:33408. doi: 10.1038/s41598-025-18761-w (PMC12479863; doi:10.1038/s41598-025-18761-w)
Supplement: Supplementary file 7 — Supplementary Material 7 [file 41598_2025_18761_MOESM7_ESM.docx]

**Supporting Information**

Figure S1. Principal component analysis.

Table S1. The primer sequences of DEGs by qRT-PCR analysis.

Table S2. Quality control for sequencing data.

Table S3. GO terms were significantly enriched in the 192 common DEGs in longan fruit during storage.

Table S4. KEGG analysis of 192 common DEGs in longan fruit during storage.

Table S5. The expression of DEGs related to sucrose metabolism and amino acids metabolism.
